# *Pseudo-nitzschia* Challenged with Co-occurring Viral Communities Display Diverse Infection Phenotypes

**DOI:** 10.3389/fmicb.2016.00527

**Published:** 2016-04-20

**Authors:** Michael C. G. Carlson, Nicolette D. McCary, Terence S. Leach, Gabrielle Rocap

**Affiliations:** School of Oceanography, University of WashingtonSeattle, WA, USA

**Keywords:** diatom, phytoplankton, *Pseudo-nitzschia*, virus, microdiversity, titer, harmful algal bloom

## Abstract

Viruses are catalysts of biogeochemical cycling, architects of microbial community structure, and terminators of phytoplankton blooms. Viral lysis of diatoms, a key group of eukaryotic phytoplankton, has the potential to impact carbon export and marine food webs. However, the impact of viruses on diatom abundance and community composition is unknown. Diatom-virus dynamics were explored by sampling every month at two coastal and estuarine locations in Washington state, USA resulting in 41 new isolates of the pennate diatom *Pseudo-nitzschia* and 20 environmental virus samples. We conducted a total of 820 pair-wise crosses of the *Pseudo-nitzschia* isolates and viral communities. Viral communities infected *Pseudo-nitzschia* isolates in 8% of the crosses overall and 16% of crosses when the host and viral communities were isolated from the same sample. Isolates ranged in their permissivity to infection with some isolates not infected by any viral samples and others infected by up to 10 viral communities. Isolates that were infected by the most viral communities also had the highest maximum observed viral titers (as high as 16000 infectious units ml^-1^). Titers of the viral communities were host dependent, as titers for one viral sample on eight different hosts spanned four orders of magnitude. Sequencing of the *Pseudo-nitzschia* Internal Transcribed Spacer 1 (ITS1) of the revealed multiple subgroups of hosts with 100% ITS1 identities that were infected by different viral communities. Indeed, we repeatedly isolated groups of isolates with identical ITS1 sequences from the same water sample that displayed different viral infection phenotypes. The interactions between *Pseudo-nitzschia* and the viral communities highlight the diversity of diatoms and emphasize the complexity and variability of diatom-virus dynamics in the ocean.

## Introduction

In the ocean, viral infection links microbial community structure, biogeochemical cycling, and microbial evolution (Breitbart, 2012). Viruses regulate marine phytoplankton communities by impacting host abundance and diversity through cell lysis (Weitz and Wilhelm, 2012). Viruses and their hosts are thought to cycle dynamically, with encounter rates favoring infection of dominant microbial taxa, which are removed due to lysis and then supplanted by new microbial populations that fill the vacant ecological niche (Thingstad, 2000). These ‘Kill-the-Winner’ dynamics have important, but often cryptic, scales of interaction and are thought to occur at varying temporal, spatial, and taxonomic levels (Thingstad, 2000; Thingstad et al., 2015). Understanding the scales of host-virus interactions is critical for accurately quantifying viral contributions to microbial mortality.

Host permissivity to viral infection and viral host range are important mechanisms that underlie kill-the-winner dynamics and directly affect the success of viruses in the ocean. Hosts with increased resistance to viral infection could outcompete other microbes with lower viral resistance by reducing viral mortality (Avrani and Lindell, 2015). Similarly, viruses may increase their chances of infection by being able to infection a broader range of hosts and thus sustain their populations. Cultured marine host-virus systems suggest that viruses range from generalists to specialists, while hosts range in their susceptibility to viral infection from highly permissive to resistant; the hierarchical ordering of these properties in hosts and viruses is known as nestedness (Flores et al., 2011). However, these traits of resistance and host range in hosts and viruses are in constant co-evolvution (Avrani et al., 2011) and thus spatial or taxonomic distance may impose barriers on host-virus interactions, called modules (Weitz et al., 2013). The patterns of nestedness and modularity can be statistically tested and have been observed in wild host-virus communities (Flores et al., 2011; Weitz et al., 2013). Phage isolated from a transect across the Atlantic were most infective of co-occurring host bacteria and formed modules driven, in part, by geographic separation (Flores et al., 2012). Waterbury and Valois (1993), when challenging *Synechococcus* isolates with environmental viral communities, demonstrated that *Synechococcus* phage titers over 2 years at the same location were not inversely correlated with *Synechococcus* abundance and thus were unimportant in controlling co-occurring cyanobacteria populations. These divergent results may be due to the small sample sizes of isolation based studies and the timing of host population cycling: isolated hosts may be in the process of being removed by their co-occurring viruses, or they may represent the supplanting microbial population that is resistant to the dominant viruses in the water. Thus, co-occurring resistance and susceptibility fluctuate in Kill-the-Winner dynamics such that both scenarios are plausible.

The dramatic boom and bust lifestyles of eukaryotic phytoplankton pose both challenges and opportunities for viruses. Eukaryotic phytoplankton blooms reach high cell densities and are often composed of few species, which may be excellent conditions for viral infection (Brussaard, 2004; Armbrust, 2009). Viral termination of blooms has been observed in eukaryotic phytoplankton–virus systems such as *Emiliania, Phaeocystis, Heterosigma, Aureococcus*, and *Micromonas* (Bratbak et al., 1993; Cottrell and Suttle, 1995; Tarutani et al., 2000; Gobler et al., 2004; Baudoux et al., 2006; Vardi et al., 2009; Lehahn et al., 2014). Under non-bloom conditions, viruses of eukaryotic phytoplankton must survive times of host scarcity since the propagation of viruses relies on contact rates between hosts and viruses. Viruses may rely on alternative strategies of propagation such lysogeny and latent infections (McDaniel et al., 2002; Thyrhaug et al., 2003), or sequestration in sediments (Tomaru et al., 2011) during these times. Ultimately, the reduction of viral abundance during times of host scarcity may be a mechanism that eventually allows phytoplankton to increase in abundance without immediate infection by viruses.

Diatoms are a group of diverse and ubiquitously distributed eukaryotic phytoplankton that exemplify the “bloom and bust” lifestyle. They dominate primarily in temperate coastal and polar oceans where they can form massive blooms, which fuel carbon export and productive food webs (Nelson et al., 1995). *Pseudo-nitzschia* is a cosmopolitan genus within the diatoms consisting of 37 described species (Lelong et al., 2012; Trainer et al., 2012). *Pseudo-nitzschia* is particularly known for the ability to produce the neurotoxin domoic acid, which can be biomagnified through food webs and can disrupt ecosystems and create public health concerns (Bates et al., 1989; Scholin et al., 2000). Toxin production varies by species (Trainer et al., 2012), underscoring the importance of *Pseudo-nitzschia* community structure for understanding toxic bloom formation.

The first diatom viruses were isolated and characterized only a decade ago (Nagasaki et al., 2004) and since then the number of diatom viruses has grown to 15, isolated on 4 genera, the centric diatoms *Rhizosolenia*, *Chaetoceros*, and the pennate diatoms, *Asterionellopsis* and *Thalassionema* (Bettarel et al., 2005; Eissler et al., 2009; Kimura and Tomaru, 2015). All diatom viruses have fallen into two groups based on their nucleic acid content, either single stranded RNA or single stranded DNA. This is in contrast to the majority of model eukaryotic phytoplankton – virus systems that involve large double stranded DNA viruses, primarily from the Phycodna- and Megaviridae families (Nagasaki and Bratbak, 2010; Moniruzzaman et al., 2014). Furthermore, the host ranges of diatom viruses are narrow. Only a few diatom viruses, such as CdebDNAV and RsRNAV, have been shown to infect multiple hosts, all within the same species (Nagasaki et al., 2004; Tomaru et al., 2008; Kimura and Tomaru, 2015).

Thus diatom viruses are genomically and functionally different than viruses that infect other photosynthetic marine eukaryotes, while diatoms exhibit boom-and-bust dynamics similar to other photosynthetic eukaryotes. It is an open question whether the dynamics between diatoms and their viruses are also similar in their capacity to control diatom populations and terminate blooms. Diatom viral infectivity based on titers of virus concentration performed on one strain of *Chaetoceros gracilis* fluctuated seasonally, reaching a maximum during the early spring when treated with environmental viral communities from Chesapeake Bay (Bettarel et al., 2005). Similarly, viral infection of one strain of *C. tenuissimus* consistently peaked in the late summer and fall from water and sediment samples taken from coastal Japan (Tomaru et al., 2011). Additionally, Tomaru et al. (2011) designed qPCR primers that were specific to *C. tenuissimus* and *C. salsugineum*, both of which have been used to isolate viruses (Nagasaki et al., 2005; Shirai et al., 2008). Host and environmental virus abundance were monitored over the span of several years with qPCR and titers, however, no correlation between fluctuations in the *Chaetoceros* species abundance and virus abundance was found (Tomaru et al., 2011). With only three diatom cultures assessed for viral titers in the field, the scales at which diatom and virus dynamics operate are still not well understood.

In this study, we assessed the temporal and spatial scales of diatom–virus interactions and quantified patterns of infection and host permissivity using the toxic diatom *Pseudo-nitzschia*. New cultures of *Pseudo-nitzschia* were isolated and crossed with environmental viral communities taken every month from two locations in the Pacific Northwest, similar in approach to cultured virus-host systems but with a mixture of wild viruses. The resulting patterns in infectious crosses between *Pseudo-nitzschia* and members of the environmental virus communities were used to understand how viral infection changed between locations and in time, how infection patterns correlated with host genotype, and how these interactions might shape *Pseudo-nitzschia* communities in the field.

## Materials and Methods

### Environmental Virus Sample Collection and Purification

**FIGURE 1:**
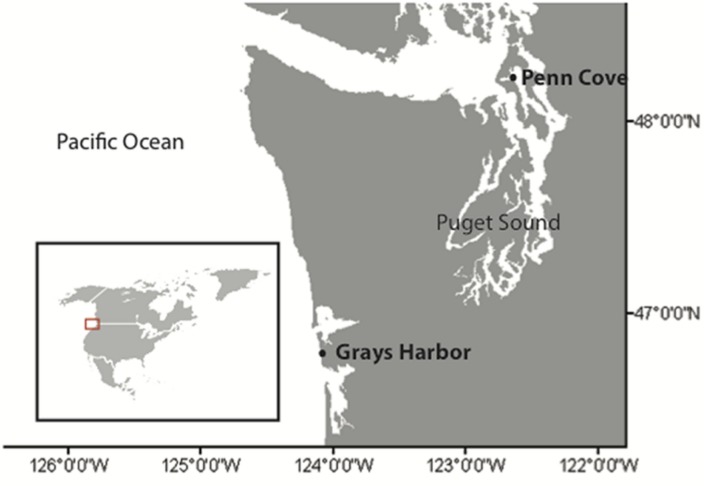
**Locations of sampling.** Penn Cove, located in the Puget Sound estuary, and Grays Harbor located on the coast of Washington state, USA. Inset map of North America shows the region of sampling.

Samples were collected at Penn Cove, Washington (48.2397, -122.6795) and Grays Harbor, Washington (46.7625, -124.0898) (**Figure 1**) monthly from April 2013 to April 2014 except from November to February, when sampling occurred at Penn Cove in November and January and at Grays Harbor in December and February. Approximately 15 L of surface water was filtered through triplicate 3.0 and 0.2 μm 147 mm polyethersulfone filters (Millepore) with a peristaltic pump within 2 h of sampling. The filters were frozen at -80°C for molecular analysis. The viruses in the filtrate were precipitated by adding iron chloride (1 g L^-1^) and incubating for 12 h at 13°C in the dark (John et al., 2011). The iron-precipitated viruses were collected by filtering on to 2.0 and 0.2 μm 147 polycarbonate filters (Millepore). The viruses were resuspended from both filters by incubating them with 0.2 M ascorbate-0.25 M EDTA-Mg2-0.25 M Tris-HCL for at least 24 h with periodic shaking (John et al., 2011). The resuspended viruses were purified to remove Fe-EDTA complexes by adding 10% poly ethylene glycol (PEG) and 2% NaCl final concentrations and incubating at 4°C for 24 h. The samples were then spun at 12000 × g for 20 min at 4°C to pellet the viruses. The supernatant was removed and the pellet washed three times with modified SM buffer (0.4 M NaCl, 0.02 M MgSO_4_, 0.05 M Tris, pH 7.5). The pellet was resuspended in 2 ml SM buffer, spun, and again washed three times with modified SM buffer. The final pellet was resuspended in 20 ml SM buffer. The PEG was removed according to Colombet and Sime-ngando (2012) by adding 1 M KCl and incubating the samples on ice for 20 min. The solution was centrifuged at 8000 × *g* for 10 min at 4°C and the supernatant containing the viruses was removed. The purified virus concentrates were stored at 4°C.

### *Pseudo-nitzschia* Community Analysis

Subsections of the 3.0 μm filters collected during sampling events were extracted using a Qiagen DNeasy minikit. The Internal Transcribed Spacer 1 (ITS1) was amplified for Automated Ribosomal Intergenic Spacer Analysis (ARISA) using triplicate PCR amplifications of the purified DNA of environmental DNA or culture DNA using primers PnALLF and 6FAM-PnALLR for 34 cycles with conditions described in Hubbard et al. (2008). Reactions were purified with a Qiaquick PCR Purification Kit, and sequenced at the University of Washington Fred Hutchinson Research Center. Two blanks, one water and one negative control from the ARISA PCR, were used to establish a noise baseline for each run. ARISA reactions were confirmed to be in linear amplification phase at cycle 34 for semiquantitive analysis using iQ SYBR green supermix (BioRad) with the same DNA and primer concentrations and cycling conditions as the reactions above.

Electropherograms of the ARISA runs were analyzed using PeakScanner (Life Technologies). Peaks were called if they were above a 50:1 signal to noise ratio, between 120 and 300 bp in length, and represented at least 3% of the total fluorescence. Peak area was summed over two base pair bins and divided by total fluorescence, which gave a relative abundance. Peaks were identified using predicted ITS lengths from the *Pseudo-nitzschia* isolates as well as from reported ITS lengths in Hubbard et al. (2008, 2014). The resulting community profiles were analyzed using the statistics package Primer-6 (Clarke and Warwick, 2001). Similarity between community profiles was calculated using both Bray–Curtis and Jaccard matrices. Matrices were clustered and tested for significance using a SIMPROF test.

### Isolation and Identification of *Pseudo-nitzschia*

A 10 μm net was hand-towed through the water for approximately 5 min during each sampling event. Single chains of *Pseudo-nitzschia* were picked with a pipette and purified through three washes with f/20 medium. All cultures in this study were maintained in f/2 medium + silica at 13°C at an irradiance of 26.7 μmol photons m^-2^ s^-1^ with cool white fluorescent illumination on a 16:8 light-dark cycle. Isolated strains were verified by light microscopy to be unialgal but not axenic.

Cultures were grown to mid-exponential phase and centrifuged at 5000 × *g* for 5 min to pellet cells. DNA was extracted with a DNeasy plant minikit (Qiagen) according to the manufacturer’s protocol. The ITS1 amplification was based on the methods in Hubbard et al. (2008). PCR primers Euk-18SF and Euk-5.8SR were used to amplify the full-length ITS1 sequence of the *Pseudo-nitzschia* strains. PCR amplicons were purified with Qiaquick PCR Purification Kit and sequenced using Euk-18SF and Euk-5.8SR primers with Sanger sequencing at Genewiz (Seattle, WA, USA) and University of Washington High Throughput Sequencing Center (Seattle, WA, USA). Sequences were identified taxonomically based on greater than 97% sequence identity to sequences of scanning electron micrograph (SEM) verified cultures in GenBank. MUSCLE (Edgar, 2004) was used for alignments and pairwise percent identities calculations. Sequences have been deposited in GenBank under accession numbers KR053126-KR053164.

### Crosses of *Pseudo-nitzschia* Isolates with Environmental Samples

*Pseudo-nitzschia* culture growth was monitored via chlorophyll-a fluorescence on a Turner AU-10 fluorometer. All experiments were conducted with cultures in mid-exponential phase. All crosses between *Pseudo-nitzschia* isolates and environmental viral communities were performed in 48 well plates (Corning) in replicates of 5 with 1 ml culture and 20 μl of purified environmental virus concentrates. For each cross, a control culture was amended with 20 μl f/2 media and a second control consisted of a culture inoculated with 20 μl of virus concentrate that was UV irradiated (100 cm from a Philips TUV 36 T5 SP UV bulb) for 15 min. Crosses were maintained under the same temperature and light conditions as described above for isolate culturing. Culture growth in well plates was measured via chlorophyll-a fluorescence on a Spectramax M2 Plate Reader (Molecular Devices). Treated wells that declined by more than half of their maximum fluorescence during the time period control cultures were still healthy were scored as infected. Concentrations of infectious units per unit volume in the viral concentrates were determined based on most probable number (MPN) tables. The range of infectious units for each infectious cross was based the number of replicates that died and the minimum and maximum infectious units that could result from the possible combinations of MPN values. Infectious units ml^-1^ of whole seawater were calculated accounting for concentration of viruses from whole water to the final viral community concentrate and assuming 100% retention of viral infectivity during filtering, flocculation, and storage.

To calculate viral titers a series of 10-fold dilutions of the environmental virus concentrates was created, with dilutions ranging from 10^0^ to 10^-7^ of the original. Each dilution was inoculated (20 μl) into 1 ml cultures of exponentially growing *Pseudo-nitzschia* in replicates of 5. Again, a control culture was amended with 20 μl f/2 media. The growth and death of the *Pseudo-nitzschia* in titer experiments was monitored as above via chlorophyll-a fluorescence. The infectious units were determined based on MPN tables, and the concentration of infectious units in seawater was calculated as described above.

### Statistical Analysis of Infection Networks

Statistical structure of the infection network generated from the crosses was tested using the BiMat package developed by Flores et al. (2016) in MatLab. Tests of modularity, using the Adaptive Brim algorithm, and nestedness, using NODF (nestedness measure based on overlap and decreasing fills) were compared to 10000 equiprobable randomized networks for statistical significance. Correlation between modules and ITS1 genotype, location, time, and infection permissivity were tested by comparing the Shannon diversity index of modules based on the predetermined categories to modules with randomly assigned categories (Flores et al., 2016).

## Results

### Environmental Conditions and *Pseudo-nitzschia* Community Structure

**FIGURE 2:**
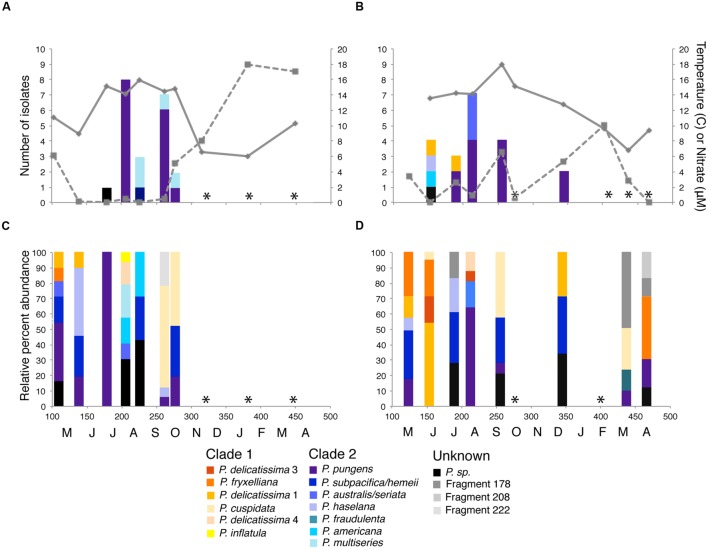
***Pseudo-nitzschia* isolates obtained and community composition at **(A,C)** Penn Cove and **(B,D)** Grays Harbor from April 2013 to April 2014.** Time of sampling is shown in Julian day and monthly increments on the *x*-axis. Solid lines are water temperature and dashed lines are nitrate concentration. *Pseudo-nitzschia* species are colored by phylogenetic clade (Lundholm et al., 2002, Guannel, unpublished data) with members of clade 1 represented by warm colors and clade 2 represented by cool colors. Unidentifiable ARISA fragments are represented in grayscale. Black bars represent ARISA fragments for isolated *Pseudo-nitzschia* with no species identification. ^∗^ indicates months with no detectable *Pseudo-nitzschia* by ARISA or in net tows.

Samples for viral communities and *Pseudo-nitzschia* isolates were taken every month from April 2013 to April 2014 (except for December and February in Penn Cove and November and January in Grays Harbor) at two sites (**Figure 1**). Penn Cove is a shallow (20 m depth) inlet in the Puget Sound estuary and Grays Harbor is located on the Pacific coast of Washington State. In total, 20 environmental virus communities were sampled and 41 *Pseudo-nitzschia* strains were isolated. *Pseudo-nitzschia* were isolated successfully during summer months when water temperatures were warm (13–17°C), nutrient concentrations were low (<6 μM NO_3_^-^) and *Pseudo-nitzschia* was abundant enough to be found in net tow samples (**Figures 2A,B**). Nitrate concentrations were positively correlated with phosphate and silicate concentrations at Grays Harbor and Penn Cove, respectively (*p* < 0.001). Only in June 2013 in Penn Cove was *Pseudo-nitzschia* a dominant member of the phytoplankton community overall.

Eight species of *Pseudo-nitzschia* were isolated and identified based on ≥97% sequence identity of the ITS1 region to SEM verified *Pseudo-nitzschia* strains: *P. pungens, P. multiseries, P. australis, P. delicatissima, P. americana, P. hasleana*, and two unknown species. *P. pungens* was isolated in 8 samples and comprised 28/41 strains isolated. The 21 isolates from Penn Cove were dominated by *P. pungens* (15 isolates) and *P. multiseries* (4 isolates) (**Figure 2A**), while the 20 isolates from Grays Harbor were also dominated by *P. pungens* (12 isolates), and to a lesser extent *P. australis* (3 isolates), and *P. delicatissima* (2 isolates) (**Figure 2B**). Two strains, *P. sp.* GH10 and *P. sp.* PC33, were unable to be assigned species identification based on their ITS1 sequence. *P. sp.* GH10 ITS1 was 88% similar to *P. seriata* and had a 138 base pair ITS1 fragment when amplified using *Pseudo-nitzschia* specific primers. The full length ITS1 region of *P. sp.* PC33 was unable to be amplified. Both isolates were confirmed via light microscopy to be *Pseudo-nitzschia*.

*Pseudo-nitzschia* community composition at the two sites was determined by ARISA targeting the *Pseudo-nitzschia* ITS1 region (Hubbard et al., 2008). *Pseudo-nitzschia* were detectable in 15 of 20 samples (**Figures 2C,D**). *Pseudo-nitzschia* communities from both Grays Harbor and Penn Cove throughout the year were composed of 3 or more species except for a monospecific bloom of *P. pungens* at Penn Cove in June (**Figure 2C**). *P. pungens* was the most common species detected in the entire dataset, present in 5 of 7 samples from Penn Cove and 5 of 8 samples from Grays Harbor. *Pseudo-nitzschia* communities did not cluster significantly by location or time.

### Infection of *Pseudo-nitzschia* Host Strains

**FIGURE 3:**
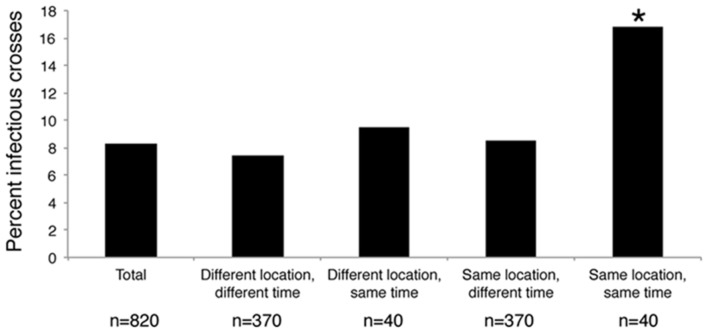
**Percent of crosses between *Pseudo-nitzschia* isolates and environmental viral communities that were infectious based on the time and location of host isolation and virus community sample collection.** ^∗^ Denotes a significant of *p*-value = 0.009 as determined by a Chi-square test.

The 41 *Pseudo-nitzschia* isolates were challenged with each of the 20 environmental virus samples in replicates of 5 to create an infection network of 820 crosses. In total, 68 *Pseudo-nitzschia* – virus community combinations (8%) showed signs of infection, defined as the death of at least one replicate in the cross (**Figure 3**). *Pseudo-nitzschia* isolates inoculated with UV irradiated viral communities showed no signs of infection compared to medium-only controls. Hosts isolated from Penn Cove were infected by virus communities from Grays Harbor, and vice versa. Hosts and viruses that came from different times and locations were not significantly less infective than the total average. Crosses of *Pseudo-nitzschia* isolates with virus communities from the same time and location resulted in infection in 16% percent of crosses, double the overall infection rate (Chi-square *p* = 0.009) (**Figure 3**).

**FIGURE 4:**
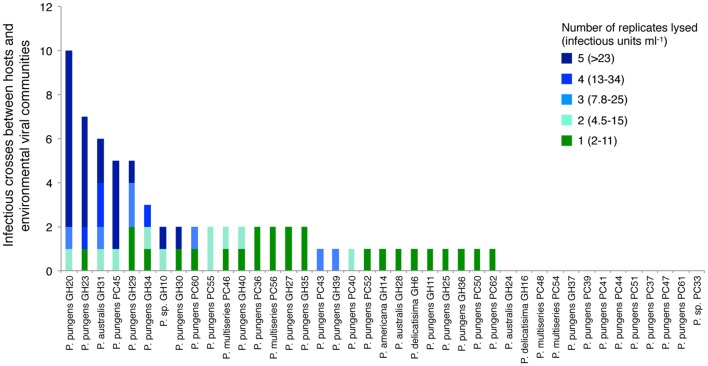
**Total number of viral community samples that resulted in an infection for each host strain.** Colors correspond to the number of replicates that were lysed and the corresponding range of infectious units based on most probable number tables for each infectious cross. Infectious units ml^-1^ of seawater were calculated assuming 100% retention of infectivity and accounting for the effect of concentrating virus from 20 L of seawater and volume of viral concentrates added to host cultures in crosses.

*Pseudo-nitzschia* isolates ranged widely in their susceptibility to infection by the viral communities (**Figure 4**). Thirteen host strains showed no detectable signs of infection from any environmental virus community. The remaining 28 strains were infected at least once, and ranged from being infected by one viral community to up to 10 viral communities. Five *Pseudo-nitzschia* hosts, *P. pungens* GH29, *P. pungens* PC45, *P. australis* GH31, *P. pungens* GH23, and *P. pungens* GH20 were infected by 5 or more viral communities, and accounted for 48% of the total infectious crosses observed. In some cases replicates displayed variable survival within one host-virus community cross, suggesting the infecting virus or viruses were present at too low a concentration to successfully infect all wells. Based on MPN calculations, bounds of infectious units ml^-1^ of whole seawater could be put on infectious crosses where between 1 and 4 replicates died (e.g., 1 replicate death = 2–11 infectious units ml^-1^, 4 replicates death = 13–34 infectious units ml^-1^) (**Figure 4**). The samples in crosses that resulted in infection in all five replicates had titers that were at least 23 infectious units ml^-1^ but the upper bound of infectious units was unknown. Hosts infected by more communities had infectious crosses suggesting more infectious units ml^-1^ (linear regression *R*^2^ = 0.536, *p* < 0.01). In the most infected host, for example, *P. pungens* GH20, all five replicates died in 8 out of the 10 infectious crosses (**Figure 4**).

### Host Specific Viral Titers

**FIGURE 5:**
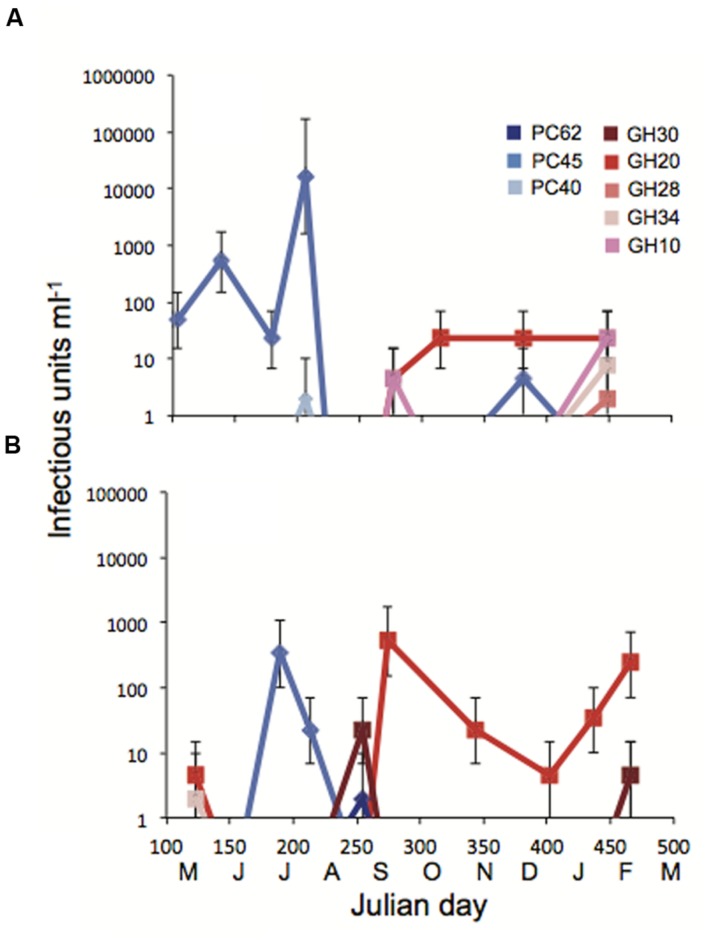
**Titers of infectious units over time in Julian days with monthly increments at (A) Penn Cove and (B) Grays Harbor.** Each of the nine strains was crossed with each of the Penn Cove or Grays Harbor viral communities. Cool colors are hosts isolated from Penn Cove, warm colors are hosts isolated from Grays Harbor. Error bars are 95% confidence intervals from 5 well MPN tables. Values below the limit of detection of 1.8 infectious units ml^-1^ are not shown.

More detailed changes in viral abundance over time were quantified by measuring titers on *Pseudo-nitzschia* strains that represented a range of susceptibilities to the viral communities. Host strains *P. pungens* PC45 and *P. pungens* GH20 were infected by 5 and 10 of the 20 viral communities respectively, while *P. sp* 1 GH10, *P. pungens* GH30, and *P. pungens* GH34 were infected by 2 or 3 of the 20 viral communities and *P. pungens* PC62, *P. pungens* PC40, and *P. australis* GH28 were each infected by a single viral community. Viral titers were determined for each of these nine hosts with every viral community. Measures of viral abundance varied by time and by host (**Figures 5A,B**). Abundance of viruses infecting host strain *P. pungens* PC45 was high, with three occurrences of above 300 infectious units ml^-1^ of whole seawater, all in summer months. The highest viral infectivity of over 10^4^ infectious units ml^-1^ seawater was observed on this strain crossed with the July Penn Cove viral community (**Figure 5A**). Interestingly, PC45 was isolated from the same water sample. However, strain *P. pungens* PC40, isolated at the same time from the same water sample as PC45, had four orders of magnitude lower viral abundance when crossed with the same July Penn Cove virus community (**Figure 5A**). This viral community did not infect the other six host strains on which titers were performed. In contrast to the high viral titers on PC45 in the summer at both locations, strain *P. pungens* GH20 had the highest titers in the fall and winter months at both locations. Host strains PC45 and GH20 that were infected by a high number of viral samples (**Figure 4**) also had the highest maximum observed titers, 16000 and 540 infectious units ml^-1^, respectively, compared with less infected strains like PC40 and GH28, which had lower maximum titers, 2 and 7.8 infectious units ml^-1^ respectively.

### Patterns of Viral Infection by Host Genotype

**FIGURE 6:**
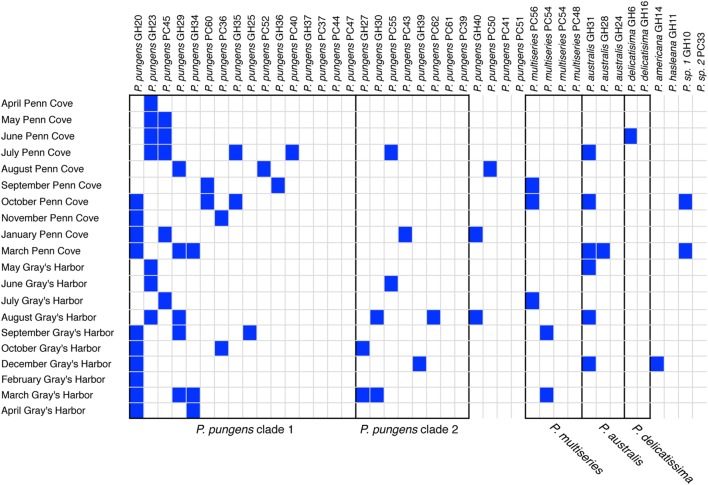
***Pseudo-nitzschia* – virus infection network.** Filled boxes represent infectious crosses. Black outlines delineate groups of hosts that share identical ITS1 sequences, which are labeled underneath.

The *Pseudo-nitzschia* hosts were grouped by ITS1 based species identification and ITS1 percent sequence identity, and ordered within each group according to the number of infectious crosses with the viral communities (**Figure 6**). Five groups of isolates had 100% nucleotide identity at the ITS1 region (**Figure 6**). Sixteen *P. pungens* strains with 100% identical ITS1 sequences consisted of 12 infection phenotypes, defined as the pattern of infection resulting from crosses with the viral communities. A second group of 8 *P. pungens* strains with a different ITS1 sequence consisted of six infection phenotypes. The phenotypes ranged from infected by multiple viral communities to not infect at all. This same pattern of diverse infection phenotypes within groups of isolates with 100% identical ITS1 sequences was observed in *P. multiseries* (three infection phenotypes in four strains), *P. australis* (two infection phenotypes in three strains), and *P. delicatissima* (two infection phenotypes in two strains) (**Figure 6**). In fact, of the 28 strains that were infected by at least 1 viral community, only two strains, *P. pungens* GH 14 and *P. americana* GH39, displayed the same infection phenotype, and they belonged to different species.

The interactions between the *Pseudo-nitzschia* and the viral communities were tested to see if there were statistically significant patterns of nestedness and/or modularity by comparing patterns in the host-virus network to 10^5^ randomized equiprobable null models. First, the network was anti-nested [nestedness value (NODF) = 0.1023, *z*-score = -3.4193, percentile = 99.83, **Supplemental Figure S1A**]. Nestedness values range from 0 to 1, with 1 representing a maximally nested network and 0 representing an anti-nested network. *Z*-scores indicate the significance of the nested pattern with values >1.96 or <-1.96 signifying statistical significance at the 5% error level (Flores et al., 2016). Finally, percentile values are the percent of the 10000 randomized networks that are more nested than the original. Anti-nested patterns are when interactions are absent from richer communities compared to less rich ones. While the *Pseudo-nitzschia* hosts sequentially increase in the number of interactions, the viral communities do not, whereas in nested patterns both viruses and hosts increase in the number of their interactions. Second, the interactions between hosts and viruses occurred in modules [modularity value (Adaptive Brim (Qb) = 0.5133, *Z*-score = 2.3491, percentile = 0.95, **Supplemental Figure S1B**], which are groups of hosts and viral communities that only infect one another. The Qb score indicates how many interactions between viral communities and hosts fall within modules. The *z*-score and percentile represent the statistical significance of the modular pattern compared to the randomized models. Interactions within modules did not group by location, time of sampling, ITS genotype, or host permissivity.

## Discussion

### Host Specific Viral Interactions

*Pseudo-nitzschia* strains ranged in their susceptibility to the viral communities sampled in this study with some hosts showing no signs of infection from any of the viral communities tested while others were infected by multiple communities. Hosts that were susceptible to more viral communities had higher maximum observed titers than less infected strains (**Figures 4** and **5**). The use of different hosts gave widely different viral titers. For example, viral titers for the July Penn Cove viral sample ranged from over 16,000 infectious units ml^-1^ to below the limit of detection depending on the host (**Figure 5A**). These titer values represent the assumption there was no loss in viral infectivity during sample concentration, in part, because no data exist on the percent recovery of single-stranded RNA or DNA viruses from seawater, only dsDNA phage (John et al., 2011). Thus these values are likely an underestimation of viral infectivity. *Prochlorococcus* and its phage exhibit similar trends of differential susceptibility and titers by host, which are the result of different host specificities of infecting viruses (Dekel-Bird et al., 2015). Furthermore, different hosts enable the isolation of different viral assemblages (Dekel-Bird et al., 2015), underscoring the need for isolating viruses on a range of hosts in order to capture a better picture of viral diversity. Together, these results highlight the difficulty of quantifying the impact of viral infection in marine systems, as investigations using cultured hosts give an incomplete picture of the natural viral community.

The patterns of host specific interactions seen in the viral infectivity or titers did not follow host genotype determined by ITS1 sequence, as strains with identical ITS sequences displayed widely varying infection phenotypes (**Figure 6**) and titers (**Figure 5**). Thus, this commonly used marker for community composition does not accurately represent the diversity with respect to viral susceptibility. Similarly, isolated diatom viruses have been observed to infect some strains but not others within one species (Nagasaki et al., 2004; Tomaru et al., 2008; Kimura and Tomaru, 2015). On nine occasions we obtained multiple isolates from the same water sample with identical ITS sequences that displayed different viral infection phenotypes. For example the *P. pungens* dominated community in August at Grays Harbor, was composed of at least four different host phenotypes that were indistinguishable based on ARISA fingerprinting or ITS1 sequencing. Diatom communities, even during blooms, are composed of populations of cells that are genetically distinct at microsatellite loci but nearly identical at the 18S, 5.8S, and ITS1 regions (Rynearson and Armbrust, 2005). Our results suggest that diatom communities are also composed of multiple coexisting diverse infection phenotypes. If multiple infection types are also present during bloom events, such diversity may impede viral termination of blooms.

Viruses have been implicated as an important factor in controlling populations of eukaryotic phytoplankton in Kill-the-Winner dynamics. Blooms of phytoplankton represent a magnified view of these dynamics, and in systems such as *Micromonas, Emiliania, and Phaeocystis*, viruses have been reported to terminate the dominant phytoplankton species (Bratbak et al., 1993; Evans et al., 2003; Baudoux et al., 2006; Vardi et al., 2009). *Pseudo-nitzschia* hosts were more likely to be infected by co-occurring viral communities. In July in Penn Cove, the co-occurring viral community and host PC45 yielded high viral titers, but low viral titers on a host of the same species PC40 also isolated from the same water. This suggests that even if PC45 was the dominant member of the bloom and viruses eliminated it, the bloom might continue because a different subpopulation of hosts similar to PC40 might replace it. Tomaru et al. (2011) over the coarse of 3 years looking at *Chaetoceros*-virus dynamics also did not find an inverse correlation between diatom abundance and viral abundance. Thus in the Kill-the-Winner model, viruses may not terminate diatom blooms as in other phytoplankton systems, but rather cycling between viruses and diatoms of the same species may be happening even during bloom events.

There are multiple mechanisms that could lead to these diverse phenotypes. Bacteria may mediate resistance to infection in diatoms, and may have played a role in the non-axenic cultures used here. For example, certain species of bacteria added to axenic cultures *Chaetoceros tenuissimus* prevented total lysis of the culture by the CtenRNAV (Kimura and Tomaru, 2014). Resistance may also be inherent to the host alone. Differential viral resistance in *Prochlorococcus* was a result of genetic diversity found in the hypervariable regions of the hosts’ genomes (Avrani et al., 2011). Also, since hosts were infected by a community of viruses, co-infection or competition between viruses could also produce numerous combinations of infection phenotypes. Predation by viruses may be stimulating phenotypic diversity in diatom communities through Red Queen dynamics where hosts and viruses are constantly evolving in response to each others changing predation strategies and defenses (Van Valen, 1973).

### Viral Community Dynamics

A major question about the ecology of diatom infecting viruses is, given the dramatic bloom and bust life style of diatoms, how are viruses propagated and successful? *Pseudo-nitzschia* communities sampled over the year became so rare that they were undetectable with ARISA 20% of the time, particularly during winter months (**Figures 2C,D**). Thus, *Pseudo-nitzschia* concentrations were likely lower than 1 cell L^-1^ (Hubbard et al., 2014). Yet the viral communities from those months were still infective of *Pseudo-nitzschia* isolates. Indeed, every virus community sample could infect at least one host. There are two explanations for this disconnect between host abundance and viral infectivity. First, the viral fraction of sediment samples has consistently been shown to be highly infectious to diatoms (Tomaru et al., 2011). The sediments could be a seed bank for diatom virus communities (Lennon and Jones, 2011). Sediment resuspension or entrainment with upwelling, which occurs during turbulent mixing events particularly in the winter and spring in the Pacific Northwest (Hickey and Banas, 2008), could be a mechanism for re-inoculating surface waters with viruses. This would allow diatom viruses to ‘overwinter’ during times of host scarcity. Second, it is possible that diatom viruses may have broader host ranges (beyond a single species) than have been detected in culture studies to date. Propagation on a wide range of hosts would allow viruses to maintain their abundance in the water column even when the concentration of one particular host was low.

Interestingly, viral communities from Grays Harbor could infect hosts from Penn Cove and vice versa (**Figure 3**). Furthermore, the rates were no different from viruses infecting hosts at the same location, but at different times. One explanation for these results may be a connectivity between viral and host populations in the Puget Sound and on the Washington coast. Based on hydrographic models of Puget Sound, surface water from Whidbey Basin, where Penn Cove is located, could reach the Washington coast in 15–30 days, while deep water from the coast would reach Whidbey on the order of 2 months at least (Babson et al., 2006). Thus, the transport of water between Puget Sound and the Washington coast occurs on temporal scales roughly similar to the frequency we sampled each individual location.

The observed infections of *Pseudo-nitzschia* were the result of the integrated infectivity of the entire viral community. Typically, virus-host networks are composed of virus isolates crossed with isolated hosts (Weitz et al., 2013), resulting in a network where both the viral isolates’ host ranges and host susceptibility is known and can then be tested for evolutionary and ecological patterns between hosts and their viruses. Here, the composition and abundance of viruses in the environmental communities was unknown. A high titer on a specific strain could be the result of many viruses at low abundance or one virus at high abundance. Similarly, the infection pattern seen in any one viral community could be due to one virus with a broad host range or many viruses with narrow host ranges. Nevertheless, the infection patterns of each viral community were highly variable from month to month. Statistical analyses indicate that the infection network is not randomly structured but is anti-nested, meaning that although hosts increased incrementally in their number of interactions with viral communities, viral communities did not display a correspondingly sequential increase in their interactions with hosts, as would be typical of nested patterns (Poulin and Guegan, 2000). Thus the hosts with multiple infections have infectious interactions with distinctly different viral communities than those with few infections. This suggests that there is high turnover in the diatom virus community rather than a resident population of viruses at both sites. Diversity in diatom viral communities could be a function of the error prone RNA-dependent RNA polymerases and rolling circle replication mechanisms used by ssRNA and ssDNA viruses, respectively, that result in high mutation rates which alter viral host ranges (Duffy et al., 2008; Nakayama et al., 2013).

## Conclusion

The patterns of virus-*Pseudo-nitzschia* interactions suggest diatom communities are extraordinarily diverse with respect to their susceptibility to viruses. Because viral infection phenotype was not correlated with host phylogeny as we can measure it with the ITS1 region, methods that estimate community composition or abundance using these markers do not capture the diversity of the community as “seen” by its viral predators. The host specific interactions can lead to large variability in infectivity and virus titers, suggesting caution should be used when interpreting titers obtained on any individual cultured hosts. The viral communities themselves changed from month to month and contained infectious members in every single sample, including those where *Pseudo-nitzschia* was not detectable. The taxonomic and temporal scales of diatom-virus interactions uncovered here illustrate the importance of permissivity and host range and emphasize the need to determine the cellular mechanisms of these attributes. Ultimately this will lead to a quantitative understanding of the impacts of viral infection on abundance and structure of wild diatom populations.

## Author Contributions

MC and GR conceived and designed the experiments. MC, NM, and TL performed the experiments and analyzed the data. MC and GR wrote the paper.

## Conflict of Interest Statement

The authors declare that the research was conducted in the absence of any commercial or financial relationships that could be construed as a potential conflict of interest.

## Supplementary Material

FIGURE S1 ***Pseudo-nitzschia* – virus infection network sorted to maximize (A) nestedness using the NODF algorithm and (B) modularity using the Adaptive Brim algorithm (Flores et al., 2016).** Columns are hosts and rows are viral communities. Curved lines represent the nested isocline below which the network would be perfectly nested. Boxes represent statistically significant modules.
