# Supplementary figures and images for: Pseudo-nitzschia Challenged with Co-occurring Viral Communities Display Diverse Infection Phenotypes
Source: Front Microbiol. 2016 Apr 20;7:527. doi: 10.3389/fmicb.2016.00527 (PMC4837327; doi:10.3389/fmicb.2016.00527)

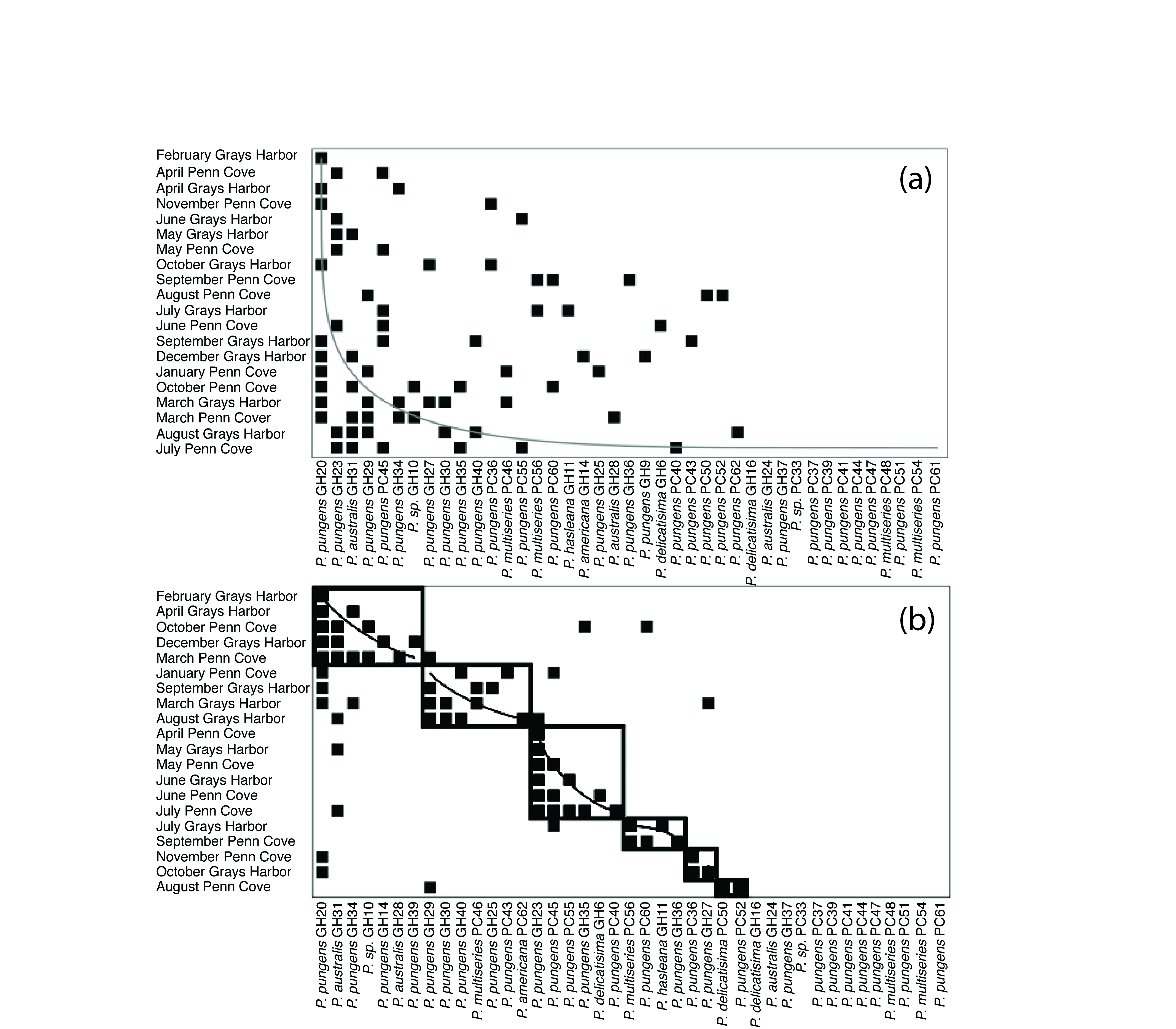

Supplement: FIGURE S1 — Pseudo-nitzschia – virus infection network sorted to maximize (A) nestedness using the NODF algorithm and (B) modularity using the Adaptive Brim algorithm (Flores et al., 2016). Columns are hosts and rows are viral communities. Curved lines represent the nested isocline below which the network would be perfectly nested. Boxes represent statistically significant modules. [file Image_1.TIF]
